# An Exploratory In Vivo Study on the Effect of Annurca Apple Extract on Hair Growth in Mice

**DOI:** 10.3390/cimb44120428

**Published:** 2022-12-09

**Authors:** Young In Lee, Seoyoon Ham, Sang Gyu Lee, Inhee Jung, Jangmi Suk, Jinhee Yoo, Su-Young Choi, Ju Hee Lee

**Affiliations:** 1 Department of Dermatology & Cutaneous Biology Research Institute, Yonsei University College of Medicine, Seoul 03722, Republic of Korea; 2 Scar Laser and Plastic Surgery Center, Yonsei Cancer Hospital, Seoul 03722, Republic of Korea; 3 Global Medical Research Center, Seoul 06526, Republic of Korea; 4 COSMAX NBT, INC, c-601, 25, Pangyo-ro 256beon-gil, Bundang-gu, Seongnam-si 18622, Gyeonggi-do, Republic of Korea

**Keywords:** annurca apple extract, hair loss, hair growth factors, cytokeratin, polyphenols

## Abstract

Hair loss is an important problem affecting the quality of life in modern society. Recent studies show that Annurca apple extract (AAE), enriched in procyanidin B2 and nutraceuticals, promotes hair growth and induces keratin production. In this study, we investigated the effects of AAE by orally administering AAE in six-week-old C57BL/6 mice once a day for 21 d. We observed improvement in hair length, thickness, weight, and density. The gene expression of two growth factors related to hair growth, vascular endothelial growth factor A (*VEGFA*) and fibroblast growth factor 7 (*FGF-7*), were measured using the quantitative reverse transcription polymerase chain reaction (qRT-PCR). The gene expression of both *VEGFA* and *FGF-7* increased significantly in the AAE-treated group. Additionally, treatment with AAE suppressed the gene expression of type 1 5α-reductase. Histological analysis showed that protein levels of cytokeratin 5 and 10 were increased in the skin tissues of the AAE-treated group. These results suggest that AAE might be a potential therapeutic natural product that prevents hair loss by promoting the expression of hair growth-related factors.

## 1. Introduction

The incidence of hair loss and thinning increases with advancing age resulting in the decline of hair density and subsequent deterioration in the quality of life in modern society. When exposed to a combination of external stress and intrinsic aging, cumulative oxidative stress can affect normal hair cycles, leading to the loss of stem cell properties and progressive hair loss. Hair density is directly linked to the maintenance of the hair cycle, which is under the control of various hormones and growth factors [1]. Among them, androgen-dependent hormonal dysregulation, typically seen in androgenetic alopecia, leads to non-scarring progressive miniaturization of hair follicles accompanied by shortening of the growth phase, called the anagen phase, in genetically predisposed men and women. Certain growth factors, such as vascular endothelial growth factor (VEGF), insulin growth factor-1, epidermal growth factor, fibroblast growth factor (FGF), noggin, wingless integration site, and keratinocyte growth factor, promote various stages of the hair cycle and have pivotal roles in hair growth [2].

Due to the increasing demand for hair growth-promoting products in the market, various nutraceuticals have been explored as potential therapeutic options for hair loss and thinning. Among them, Annurca apple extract (AAE) has shown the ability to counteract adult baldness and patterned hair loss [3]. Indigenous to Southern Italy, the Annurca apple contains a high content of oligomeric procyanidins, such as procyanidin B2, which can improve hair growth and increase hair density, weight, and keratin content [4,5].

Oligomeric procyanidins promote hair epithelial cell growth and induce the anagen phase and are the most effective natural compounds that accelerate hair growth both in vitro and in human studies [6,7,8,9] Annurca apple polyphenols stimulated the expression and biosynthesis of cytokeratins in a human epidermis model in vitro without significant interference with the regulation of dynamic cellular processes [9]. In murine epithelial cells, the active ingredient procyanidin B2 inhibits protein kinase C isozymes and modulates the expression of transforming growth factors, subsequently activating different cellular activities, including cell growth, differentiation and development [3]. A recent study involving preclinical evaluation of primary human models of follicular keratinocytes and dermal papilla cells demonstrated that in addition to antioxidant properties, AAE also increased keratin expression in keratinocytes and cytokeratin expression in the dermal papilla cells [3].

Although the effect of AAE consumption on promoting hair growth has been reported previously, its exact mechanism has not yet been elucidated. Studies have suggested that polyphenols in AAE act as antioxidants, and the metabolic shift induced by AAE prevents the oxidization of amino acids, making them available for keratin biosynthesis [4]. Although previous reports have suggested the positive role of oral AAE on hair growth based on in vitro and human clinical prospective studies [3,9], a well-designed in vivo study to reveal the effect of its oral formulation has not been performed yet.

In this study, we elucidated, for the first time, the in vivo effects of a novel oral AAE nutraceutical formulation on hair growth in C57BL/6 mice. The effect of the AAE formulation on murine hair growth was evaluated after administering it orally at two different concentrations for three weeks. In this pilot study, gene and protein expression of hair growth-related factors in the skin tissues of AAE-treated C57BL/6 mice were evaluated.

## 2. Materials and Methods

### 2.1. Experimental Animals

Five-week-old male C57BL/6 mice were obtained from the ORIENT BIO animal center (Seongnam-si, Republic of Korea). All experimental procedures were approved by the institutional animal care and use committee of Yonsei University College of Medicine (IACUC No. 2021-0234). The experiments were performed following the NIH guide for the care and use of laboratory animals. Animals were housed in cages under the following breeding conditions: temperature of 24 °C ± 0.5 °C, humidity of 55–65%, and 12 h light and 12 h dark cycle that was automatically controlled. Food and water were supplied ad libitum.

### 2.2. Experimental Design

Six-week-old C57BL/6 mice were acclimatized under constant conditions for one week. After acclimatization, the mice were anesthetized with isoflurane, and the back of the mice was shaved using an animal clipper. After applying the depilatory agent, the remaining fine hairs on the skin were removed. After 24 h of skin stabilization, the mice with telogen phase hair were randomly divided into three groups (*n* = 6): negative control group, vehicle; AAE low, 160 mg/kg/d; and AAE high, 320 mg/kg/d. The AAE used in this study was supplied form COSMAX NS(Evra S.r.l, Italy). The major active ingredient included in the test product, AAE, was procyanidin B2. A hundred microliters of each test compound was administered at the designated time every day for three weeks. After the experiment, mice were anesthetized with CO_2_ and sacrificed. RNA and proteins from the skin tissues were extracted to evaluate gene and protein expression.

### 2.3. Visual Analysis of Improvement in Hair Growth

Evaluation of differential improvements in hair growth of the three treatment groups (control, AAE low, and AAE high) was performed by visually scoring the degree of hair re-growth by three blinded investigators based on the following numerical rating scale on days 0, 7, 10, 14, 18, and 21: 0–19% (grade 1), 20–39% (grade 2), 40–59% (grade 3), 60–79% (grade 4), and 80–100% (grade 5).

### 2.4. Measurements of Hair Growth

Hair length, weight, thickness, and density were measured at the end of the three-week study. To measure the average length of hair, total hair was collected from the dorsum, and the average length of ten hair strands from each mouse was determined using Folliscope 5.0 at a magnification of × 50 (Anagen Corp., Seoul, Republic of Korea). The weight of hair was measured from 200 hair strands collected from each sacrificed mouse using an ultra-fine scale (Premium Microbalance [Cubis 2], Sartorius Corp., Seoul, Republic of Korea). To measure hair thickness and density, the dorsal skin of each mouse was extracted and fixed in 10% formalin for 24 h, and the thickness of the hair was measured by observing the skin tissue under Folliscope 5.0 (Anagen Corp., Seoul, Republic of Korea) at a magnification of 400×. The average from ten randomly selected strands from each mouse was used. The density of the hair was calculated from the same magnification using Folliscope and expressed as number per unit area (cm^2^).

### 2.5. Histological Analysis

For histological analysis, the skin tissue of each mouse was extracted and fixed in 10% formalin for more than 24 h, and then a paraffin block and paraffin-embedded slide were prepared. Deparaffinized tissues were stained with hematoxylin (104302; Merck, Kenilworth, NJ, USA) solution that stains the nucleus and washed with running water. The cytoplasm was stained with eosin (230251; Sigma-Aldrich, St. Louis, MI, USA) solution and then washed with water. The dehydrated tissues were fixed using a mounting solution. Then, the epidermis and papillary dermis of the fixed tissues were photographed at 100× magnification using an optical microscope (Olympus BX43; Olympus Co, Tokyo, Japan).

### 2.6. Quantitative Reverse Transcription Polymerase Chain Reaction (qRT-PCR)

Total RNA from skin tissues was extracted using a RNeasy RNA extraction kit (Qiagen Inc., Germantown, CA, USA) and was quantified using a NanoDrop 2000 spectrophotometer (ThermoFisher, Waltham, MA, USA). cDNA synthesis was performed using the RNA to cDNA EcoDry™ premix kit (Takara Sake, Berkley, CA, USA), according to the manufacturer’s protocol. The synthesized cDNA was amplified using TaqMan fast advanced master mix (Applied Biosystems, Waltham, MA, USA) and TaqMan primers (*SRD5a1*, Mm00614213_m1; *VEGFA*, Mm00437306_m1; FGF 7, Mm00433291_m1; Applied Biosystems) through RT-PCR, and the relative expression of each gene was normalized to that of *GAPDH* (Mm99999915_g1, Applied Biosystems), the housekeeping gene. The relative mRNA expression was calculated using the 2^−ΔΔCT^ method. The experiment was independently repeated at least three times (*n* ≥ 3).

### 2.7. Immunohistochemistry

For the evaluation of protein levels in C57BL/6 mice, the skin tissues of each mouse were collected. Tissues were fixed in 10% formalin for more than 24 h, and then paraffin blocks and paraffin-embedded slides were prepared. After deparaffinization and antigen retrieval, the tissues were incubated with primary antibodies specific to cytokeratin 5 (anti-cytokeratin 5 antibody, ab64081; Abcam, Cambridge, UK) and cytokeratin 10 (anti-cytokeratin 10 antibody, ab76318; Abcam). Polymer-horseradish peroxidase (HRP) anti-rabbit (K4003; Dako, Glostrup, Denmark) was used as the secondary antibody specific to the primary antibody. Finally, cell nuclei were stained using hematoxylin (SM806; Dako), and the tissues were fixed using a mounting solution. The fixed tissue was photographed at 400× magnification using an optical microscope (Olympus BX43; Olympus Co, Tokyo, Japan). From the tissue images, the entire area of the epidermis and the positive area of the target protein were measured using Zeiss Axioskop 2 microscope (Carl Zeiss, Inc., Jena, Germany). The region of expression of the target protein relative to the entire epidermis was expressed as the area ratio (%).

### 2.8. Statistical Analysis

All experimental data were presented as mean ± standard deviation (SD), and each experiment was conducted at least three times (*n* ≥ 3). Statistical analysis was performed using SPSS software version 25.0 (IBM Corp., Armonk, NY, USA). The statistical significance was calculated using Student’s *t*-test. Statistical significance was considered at *p* < 0.05, indicated by *.

## 3. Results

### 3.1. Hair Growth-Promoting Effect of AAE in C57BL/6 Mice

**Figure 1:**
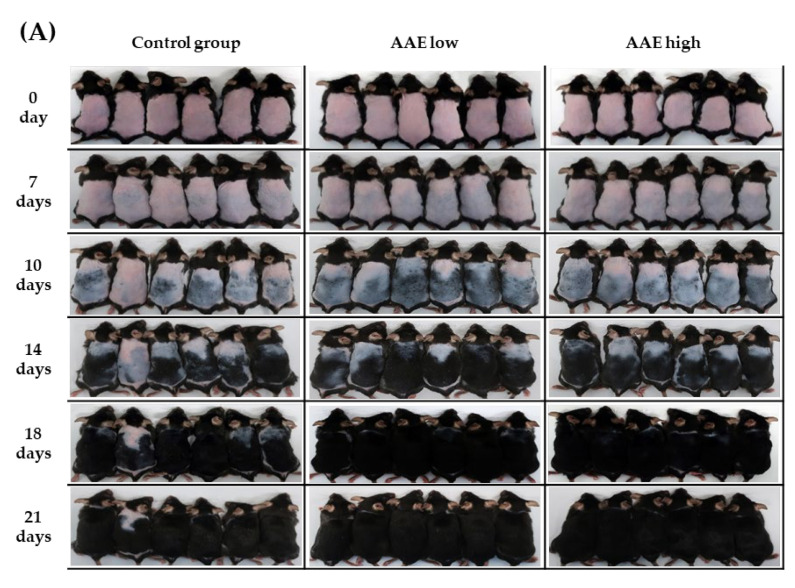

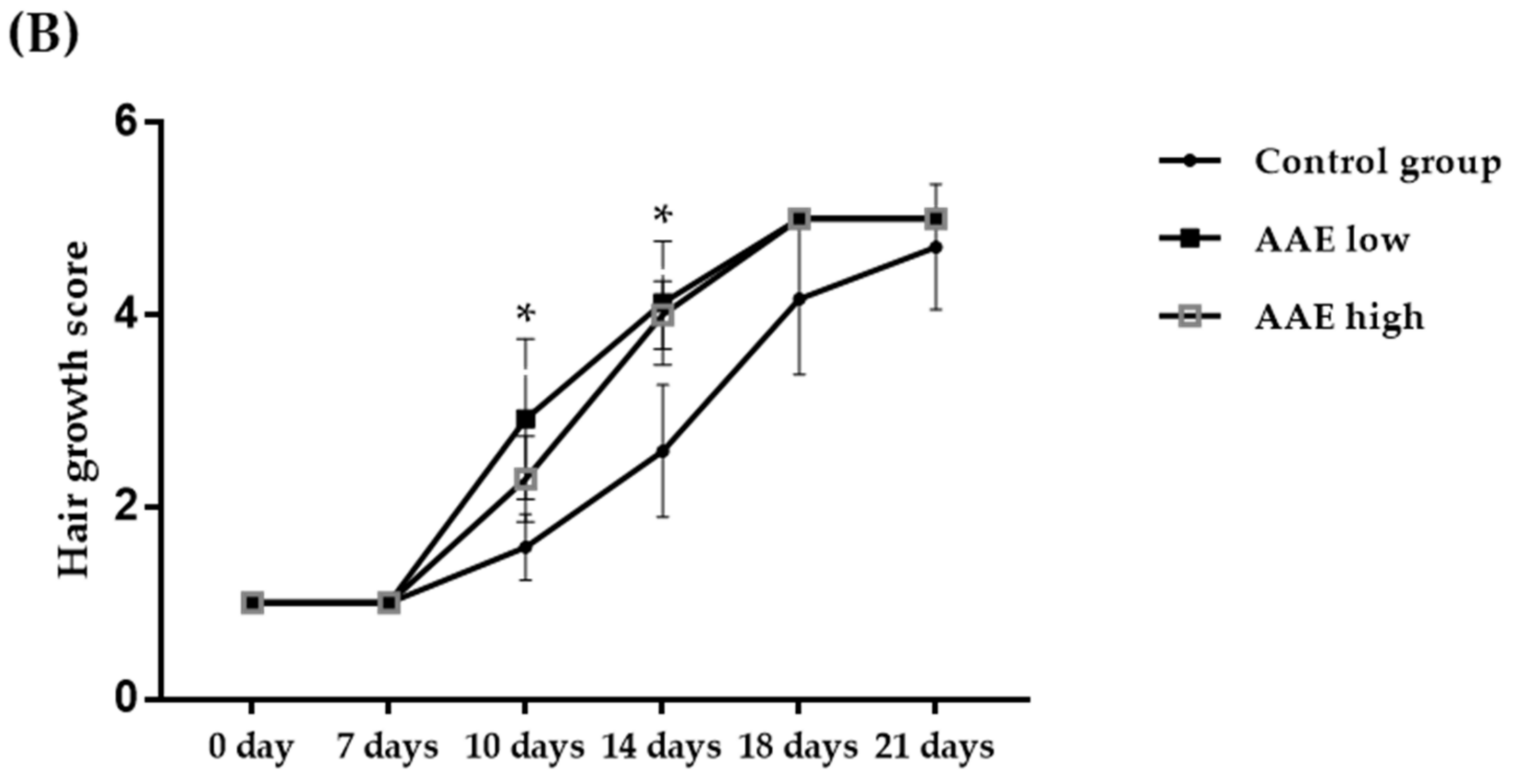
Effects of Annurca apple extract (AAE) on hair growth in C57BL/6 mice. Mice in the telogen hair phase were orally given low (160 mg/kg/d) and high (320 mg/kg/d) doses of AAE once a day for 21 d. Hair growth was measured after days 0, 7, 10, 14, 18, and 21 (**A**). Hair growth score was designated as 1, 2, 3, 4, and 5 corresponding to 0–19%, 20–39%, 40–59%, 60–79%, and 80–100% re-growth (**B**). Data are presented as mean ± SD (*n* = 6), * *p* < 0.05 compared with the control group. AAE low, Annurca apple extract at low concentration; AAE high, Annurca apple extract at high concentration.

**Figure 2:**
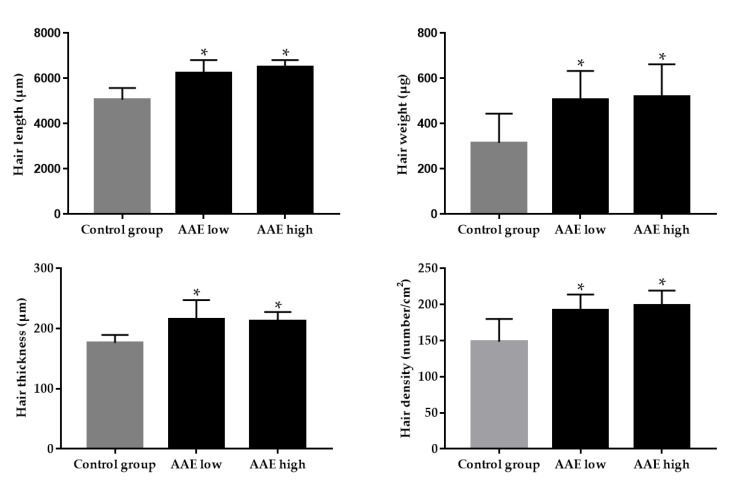
Comparison of hair length, weight, thickness, and density after three weeks of continuous treatment in the C57BL/6 mice. Both low and high AAE groups showed significantly increased hair length, thickness, weight, and density compared to the control. * *p* < 0.05 compared to the control group.

To investigate whether oral consumption of AAE has a promoting effect on hair growth in vivo, AAE was orally administered to C57BL/6 mice daily for three consecutive weeks. Eighteen C57BL/6 mice in the telogen hair phase were treated with either low AAE (160 mg/kg/d), high AAE (320 mg/kg/d), or the vehicle (control group). Each treatment group consisted of six mice. After three weeks, a visual evaluation of hair growth in C57BL/6 mice revealed that ingestion of AAE notably accelerated murine hair growth. The average improvement score of hair growth based on visual evaluation by blinded investigators was significantly higher in the low and high AAE groups than in the control group on days 10 and 14 (Figure 1, Supplementary Table S1, * *p* < 0.05). After the three-week treatment, hair length, weight, thickness, and density showed significant increases in both low and high AAE groups compared with those in the control group (Figure 2, * *p* < 0.05). Hair length, weight, and density displayed a dose-dependent increase in the AAE-treated groups.

### 3.2. Effect of AAE on Gene Expression of Growth Factors in the Skin Tissue of C57BL/6 Mice

**Figure 3:**
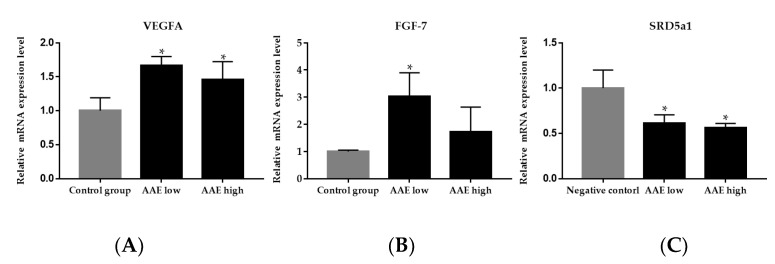
Relative mRNA expression of *VEGFA* and *FGF 7* in C57BL/6 mice. The skin tissues of mice were collected after three weeks of treatment. The expression of *VEGFA* (**A**) and *FGF-7* (**B**) were higher in the AAE-treated group compared to that in the control group. The expression of genes *VEGFA* and *FGF-7* were higher in the AAE low group (160 mg/kg/d) than AAE high group (320 mg/kg/d). Gene expression of (**C**) *SRD5a1* showed a dose-dependent decrease in AAE low and high groups compared to the control group; * *p* < 0.05 compared with the control group. *VEGFA*, vascular endothelial growth factor A; FGF 7, fibroblast growth factor 7.

To investigate the effect of AAE on the local gene expression of growth factors, we performed the quantitative reverse transcription polymerase chain reaction PCR (qRT-PCR) using RNA extracted from the dorsal skin tissue of C57BL/6 mice. We measured the expression of *VEGFA* and *FGF-7*, which are two important growth factors known to stimulate hair growth. The gene expression of both *VEGFA* and *FGF-7* increased significantly in the low and high AAE groups compared with that in the negative control group (Figure 3A,B, * *p* < 0.05).

### 3.3. Effect of AAE on Type 1 5α-Reductase Activity in the Skin Tissue of C57BL/6 Mice

To explore the effect of AAE on the local expression of the type 1 5α-reductase gene, we performed qRT-PCR using RNA extracted from the dorsal skin tissue of C57BL/6 mice after the three-week treatment regimen. The expression of type 1 5α-reductase showed a significant dose-dependent reduction in the low and high AAE groups compared to the control groups (Figure 3C, * *p* < 0.05). Our data indicated that AAE ingestion might suppress the gene expression of type 1 5α-reductase in the local skin tissue, affecting hair growth.

### 3.4. Effect of AAE on Expression of Epidermal Cytokeratins in the Skin Tissue of C57BL/6 Mice

**Figure 4:**
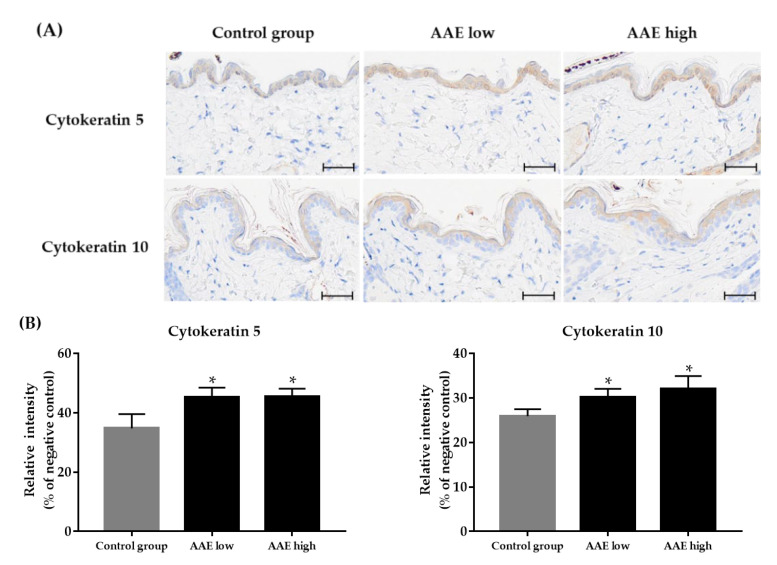
Comparison of expression of Cytokeratin 5 and 10 using immunohistochemistry (**A**) Digital photomicrographs were taken from areas at a fixed magnification of 400×. Scale bars on tissue slides indicate 20 μm. The stained area of the Cytokeratin 5 and 10, relative to the entire epidermis (%), was significantly increased in both AAE-treated groups compared to in the control (**B**). * *p* < 0.05 compared with the control group.

We performed immunohistochemical staining to study the effect of AAE consumption on local expression of epidermal cytokeratins, CK5 and CK10. The CK5 and CK10 protein levels showed a dose-dependent significant increase in the low and high AAE groups compared to those in the control group (Figure 4, *p < 0.05).

## 4. Discussion

Although the effect of AAE consumption on promoting hair growth has been reported previously, its exact mechanism has not yet been elucidated. Studies have suggested that polyphenols in AAE act as antioxidants, and the metabolic shift induced by AAE prevents the oxidization of amino acids, making them available for keratin biosynthesis [4]. Although previous reports have suggested the positive role of oral AAE in hair growth based on in vitro and human clinical prospective studies [3,9] a well-designed in vivo study to reveal the effect of its oral formulation has not been performed yet. We conducted a pilot study to investigate the effect of oral consumption of AAE on murine hair growth and revealed that it significantly decreased the local gene expression of 5α-reductase, VEGF, and *FGF-7*, as well as the local protein expression of cytokeratin 5 and 10 in skin tissue.

Compared to the negative control group with placebo, the gene expression of growth factors (VEGF and *FGF-7*) in the dorsal skin of AAE-treated mice groups were significantly upregulated. VEGF is a well-known angiogenesis-promoting growth factor that stimulates hair growth in both mice and humans. An increase in angiogenesis may accelerate anagen transition and hair follicle re-growth [10]. VEGF transgenic mice display an increased number and size of hair follicles compared to wild-type mice [11,12]. In contrast, *FGF-7* is a member of the FGF family, which promotes hair follicle proliferation, differentiation, and prolonged anagen phase [13,14,15]. *FGF-7* increases the survival and regeneration of hair follicles [16]. The dermal papilla produces *FGF-7*, whose receptors are highly expressed in the overlying matrix cells during the anagen phase [17]. *FGF-7* is also known as the keratinocyte growth factor, which plays an important role in regulating proliferation in epithelial tissue and protects the hair follicle from cytotoxic agents or UV irradiation [18].

5α-reductase converts testosterone to dihydrotestosterone (DHT), which has a stronger affinity for androgen receptors than testosterone and induces the expression of genes related to the reduction of hair follicles, which ultimately results in hair loss. Although 5α-reductase inhibitors such as finasteride have been approved for hair loss treatment, they can cause a series of notable side effects such as sexual dysfunction, depression, and gynecomastia [19]. Therefore, searching for natural compounds that have a potential inhibitory effect on the activity of 5α-reductase to promote hair re-growth is worthwhile. We performed gene expression analysis of type 1 5α-reductase, which was significantly decreased in the skin tissue of the AAE-treated group compared to the control group. These results indicate a possible role of AAE nutraceutical formulation in inhibiting the 5α-reductase activity.

One of the first published articles discussing the effect of AAE on hair growth has shown that AAE enhances keratin expression in a human model of skin and promotes hair re-growth [9]. Keratins, including epidermal cytokeratins, are fundamental in epidermal and hair development and homeostasis, as well as in hair follicle growth and maturation [20]. In this study, we discovered that AAE ingestion increases the gene expression of the keratinocyte growth factor (also known as *FGF-7*), which can promote the proliferation of epithelial tissue. We further investigated the expression of epidermal cytokeratins, including CK5 and CK10, and revealed their increased protein expressions in the skin tissues of AAE-treated groups compared to the levels in the control group. In a previous study, the consumption of AAE exerted partial effect in increasing epidermal cytokeratins, including CK5 and CK10 [3,9]. Studies have revealed that AAE stimulates production of cytokeratins, which not only shows a considerable hair-inductive activity, but also promptly improves skin quality [21]. Since keratins are known to be fundamental in epidermal and hair development and homeostasis, the regulation of keratin expression appears to be a central event for rapid proliferating epidermal cells, as well as in hair follicle growth and maturation [9]. Hence, the increased expression of epidermal cytokeratins after ingestion of AAE might protect the hair follicle from external stresses by preventing abnormal epithelial thinning, thereby promoting hair growth. Procyanidin B2, an antioxidant, is also known to stimulate the production of hair keratin. Our preliminary data demonstrated that three-week ingestion of AAE increased the hair shine as compared to that of the control, when graded visually by two blinded investigators at the end of the study (data not shown). Further controlled study on the effect of procyanidin B2 on hair shine using a gloss meter is required to substantiate our result.

Overall, our results suggest that AAE might be a potential therapeutic natural product that prevents hair loss by promoting the expression of hair growth-related factors. Especially AAE low group (160 mg/kg/d) compared with AAE high group (320 mg/kg/d) showed relatively high increases in not only the hair growth score in earlier phase of the experiment, but also the gene expression levels of growth factors, such as VEGF and FGF. Our study has several limitations. First, the sample sizes of each treatment group were limited. Secondly, a limited number of hair growth-related factors, including growth factors and cytokeratins, were analyzed in the current experimental setting. Further exploration of the expression of various genes and proteins via in vitro study is required to hypothesize a detailed mechanism underlying hair growth due to the oral consumption of AAE. One of the possible mechanisms might be the activation of β-catenin via the canonical Wnt signaling pathway. When β-catenin is activated, it moves into the nucleus to induce the transcription of growth factors, such as VEGF and FGF [22]. A well-designed in vitro study is required to establish the molecular pathophysiology of AAE in promoting hair growth.

## Data Availability

Not applicable.

## Supplementary Materials

The following supporting information can be downloaded at: https://www.mdpi.com/article/10.3390/cimb44120428/s1, Supplementary Table S1: Hair growth score for animals after three weeks of continuous treatment with AAE.
