# Supplementary material for: An Exploratory In Vivo Study on the Effect of Annurca Apple Extract on Hair Growth in Mice
Source: Curr Issues Mol Biol. 2022 Dec 9;44(12):6280–9. doi: 10.3390/cimb44120428 (PMC9776982; doi:10.3390/cimb44120428)
Supplement: Supplementary file 1 [file cimb-44-00428-s001.zip › cimb-2012628-supplementary.pdf]

Table S1. Hair growth score for 21 days of treatment with AAE.

Control Group	AAE Low	AAE High
1.000 ± 0.000	1.000 ± 0.000	1.000 ± 0.000
1.583 ± 0.000	1.000 ± 0.000	1.000 ± 0.000
1.583 ± 0.344	2.917 ± 0.837*	2.292 ± 0.443*
2.583 ± 0.687	4.125 ± 0.641*	4.000 ± 0.354*
4.167 ± 0.786	5.000 ± 0.000	5.000 ± 0.000
4.708 ± 0.652	5.000 ± 0.000	5.000 ± 0.000
